# Evidence-ranked motif identification

**DOI:** 10.1186/gb-2010-11-2-r19

**Published:** 2010-02-15

**Authors:** Stoyan Georgiev, Alan P Boyle, Karthik Jayasurya, Xuan Ding, Sayan Mukherjee, Uwe Ohler

**Affiliations:** 1 Program for Computational Biology and Bioinformatics, Duke University, 102 North Building, Durham, NC 27708, USA; 2 Institute for Genome Sciences and Policy, Duke University, 101 Science Drive, Durham, NC 27708, USA; 3 Department of Computer Science, Duke University, 450 Research Drive, Durham, NC 27708, USA; 4 Department of Statistical Science, Duke University, 214 Old Chemistry Building, Durham, NC 27708, USA; 5 Mathematics Department, Duke University, 102 Science Drive, Durham, NC 27708, USA; 6 Department of Biostatistics and Bioinformatics, Duke University, Duke University School of Medicine, 2424 Erwin Road, Durham NC 27710, USA

## Abstract

cERMIT is a computationally efficient motif discovery tool based on analyzing genome-wide quantitative regulatory evidence. Instead of pre-selecting promising candidate sequences, it utilizes information across all sequence regions to search for high-scoring motifs. We apply cERMIT on a range of direct binding and overexpression datasets; it substantially outperforms state-of-the-art approaches on curated ChIP-chip datasets, and easily scales to current mammalian ChIP-seq experiments with data on thousands of non-coding regions.

## Abstract 2

A new computational method for the identification of regulatory motifs from large genomic datasets is presented here

## Background

With the continuing growth and scale-up of genome and transcriptome sequencing of a large number of eukaryotes, there has been increasing interest in gaining a better understanding of the functional connections between all the genes within a complex organism. Regulatory factors that control the activation or repression of a gene on the transcriptional or post-transcriptional level often recognize specific DNA or RNA sequence elements. One of the first steps towards understanding the functional characteristics of regulators such as transcription factors (TFs) is to obtain accurate representations of their preferred binding sites and the location of their occurrences, which can then be utilized to identify candidate genes under direct regulatory influence of a TF. Regulatory elements tend to be short (about 6 to 15 bp in eukaryotes) and often highly degenerate, which makes it difficult to distinguish them from the surrounding sequence, which is orders of magnitude larger in size [1-3]. The task to identify a representation for a functional sequence element is commonly referred to as (*de novo*) motif finding.

The motif finding problem has been traditionally phrased as the following: Given a set of putatively co-regulated genes, find the optimal motif description and the set of occurrence locations in the corresponding regulatory regions. Many popular approaches are based on iterative updating of a position-specific scoring matrix (PSSM) representation of the binding site, which reflects the affinity of the protein to its functional sites. Stochastic searches in the form of Gibbs sampling or expectation maximization-based algorithms have been used extensively to address this goal by means of iteratively optimizing a suitable objective function [4-7]. The use of additional information, such as sequences from related species (for example, [8]), or priors on the TF binding domain or nucleosome positions [9,10], has led to noticeable improvements in the performance of these strategies. As alternatives to PSSMs, motifs can be described as consensus strings over a degenerate alphabet. This representation has allowed for the exhaustive identification of motifs that are over-represented compared to a genomic background model [11-14], and frequently makes use of efficient data structures such as suffix arrays to search for overrepresented oligomers [15,16]. This strategy places the focus directly on optimizing the motif description without having to specify an explicit generative model for the entire DNA sequence.

The detection of functional DNA motifs has been greatly facilitated by the availability of high-throughput functional genomics data that provide direct or indirect evidence for gene regulation. For instance, the genome-wide DNA occupancy by a particular TF can now commonly be measured through *in vivo *approaches such as chromatin immunoprecipitation (ChIP) followed by hybridization of fragments to microarrays (ChIP-chip) [17,18] or deep sequencing (ChIP-seq) [19]. Such experiments have been shown to regularly identify hundreds or thousands of enriched regions for individual factors. However, some of the most popular existing approaches scale badly and are computationally infeasible when applied to sets with thousands of candidate regulatory sequences. For instance, the sampling step in PSSM-based approaches is typically performed on the positions of the regulatory sequences, and samples are then used to update the motif model. Due to these limitations, existing approaches have often used genome-wide quantitative data only to reduce the search space. This is particularly the case for the runtime extensive sampling based methods, which have thus been applied on a subset of high-scoring or otherwise pre-filtered regions [20], or have additionally used low-scoring sequences as 'discriminative' evidence to direct the search [21].

Instead of modifying traditional approaches, the availability of qauntitative data suggests the possibility for an alternative definition of the motif finding problem: identify enriched sequence motifs, given quantitative experimental evidence for a genome-wide set of regulatory regions. This formulation allows one to explicitly utilize the total quantitative information from the experiment, rather than to only use it to define a set of promising target sequences, and then proceed with motif finding as usual. The motif finder REDUCE [22] was an early exponent of this framework, and applied a linear regression strategy to fit the log expression ratios from microarray experiments to the sum of contributions from a set of putative regulators. This promising approach was later followed up with MatrixReduce [23], which is based on a non-linear statistical mechanics model of TF-DNA interactions fitted to ChIP-chip data. A common feature of approaches in this category is that all the experimental data are used in the model, avoiding the use of an explicit significance threshold. In addition, the utilization of all probes from the high-throughput experiment generally does not require an explicit model for background sequence. Falling between the strategy to integrate the complete quantitative data, and the above-mentioned approaches that use only the top sequences based on pre-defined cutoffs, recent studies have also attempted to explicitly infer optimal cutoffs that distinguish positive from negative probes. Examples include DRIM [24] and Amadeus [25], both of which are based on simple hypergeometric distribution-based criteria.

Here, we propose a new motif identification system, the (conserved) evidence-ranked motif identification tool, or cERMIT, which makes use of the complete data without the need to pre-define or infer thresholds. It is explicitly designed to be able to analyze current large genomic regulatory datasets such as those from ChIP-chip or ChIP-seq experiments, and we demonstrate its superior performance on gold-standard high-throughput ChIP-chip datasets. We have integrated cERMIT as the final step in a pipeline for motif inference on ChIP-seq datasets, which includes the alignment of high-throughput sequencing reads [26] and peak calling of enriched locations [27], and utilizes genome-wide information on open chromatin as determined by DNaseI hypersensitive assays. Finally, we demonstrate its wide applicability by an analysis of miRNA overexpression experiments.

## Results

### Overview

In a nutshell, cERMIT takes putative regulatory regions *S *and scores representing evidence of direct or indirect regulation as input *E *and searches for an optimal motif of flexible length, represented as a degenerate consensus sequence over the IUPAC alphabet. A post-processing step allows generation of PSSMs from high scoring candidates.

**Figure 1:**
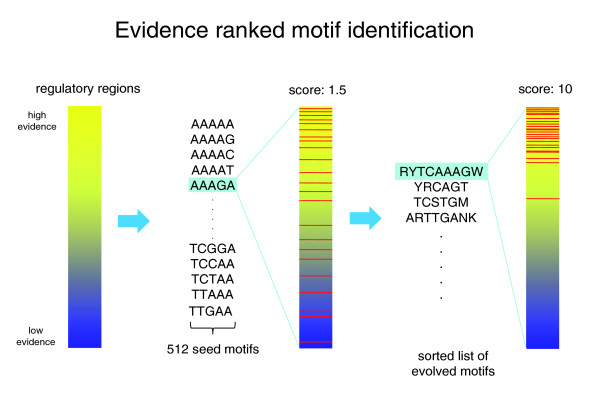
**cERMIT motif discovery algorithm**. cERMIT starts with all possible 5-mer seeds and proceeds by independently 'evolving' each seed by increasing the enrichment of target sequences in the top of the evidence ranked list.

The objective function we use to score motif candidates is inspired by gene set enrichment analysis [28-30] and encapsulates the aggregate evidence of regulation for a set of sequence regions. More specifically, we summarize the group evidence of binding by a centered and scaled average relative to a random group of the same size. It is used to search for the best partition of *S *into candidate positive and negative sets, where the positive set consists of regions that have at least one occurrence of a candidate motif, while the negative set contains the remaining sequence regions in *S*. We begin the search from the comprehensive set of all possible non-degenerate five-mers, each of which defines an initial starting partition of *S*. Each of the five-mers is then 'evolved' in a greedy search by varying motif length or degeneracy (Figure 1). cERMIT can take different data as evidence for regulatory interactions, and can optionally utilize orthologous sequences from related species to restrict the search to co-occurring motifs.

For a controlled evaluation of a new motif discovery approach, it is desirable to have reliable sets of positive examples for which it is straightforward to compare the success of different strategies. ChIP-chip or ChIP-seq data on factors with known literature binding site consensus sequences provide the most straightforward setting, as they imply direct evidence of binding, presumably mediated by a common sequence motif. To provide a common ground with other recent algorithms, we focus on the genome-wide yeast ChIP-chip dataset from the Young lab, which is still the most comprehensive ChIP dataset [31], but also demonstrate the application of cERMIT on a compendium of recent mammalian ChIP-seq datasets. Finally, we consider microarray and mass spectrometry data collected from microRNA overexpression experiments to show that the motif finder performs well in cases where the influence of a factor is not determined by a direct binding assay but rather by downstream changes in mRNA or protein expression levels.

### Elucidating regulatory sequence from ChIP-chip experiments

The TF dataset from [31] consists of genome-wide location data for 203 yeast TFs assayed in a total of 352 different experiments; 82 TFs were assayed in more than one condition. The input consists of an upstream sequence for each gene, as well as an associated *P*-value of binding of a specific TF to each upstream sequence. Previous studies [31,32] have combined known literature consensi with the results of different motif finders to arrive at a comprehensive list of binding site representations. Knowing the literature consensus provides us with a common basis to compare the performance of motif finders, but different publications use different criteria to define success. We here use the PSSM similarity metric introduced by [31] (see Equation 6 in the Materials and methods section). Varying the similarity threshold cutoff will of course influence the absolute number of successful predictions, but for any fixed cutoff, it provides a relatively fair assessment of different algorithms. We chose cutoff values reported in previous evaluations on the same dataset.

The yeast dataset has been used as a starting point for many recent motif finder evaluations, of which we will use two to assess our new approach. While yeast is often regarded as 'easy' with respect to regulatory sequence analysis, these assessments demonstrated that there was still considerable room for possible improvement. The first evaluation focused on a subset of 156 out of the 352 total experiments for which there was strong evidence of more than 10 bound probes (*P*-value < 0.001) [10]. This gold standard set for motif finders covers 80 unique TFs for which there is a known literature consensus binding site [32]. With the idea that a ChIP experiment should strongly enrich for sequences sharing the binding site of the TF assayed, a motif was only counted as successfully identified if the top prediction matched the known consensus at a cutoff of 0.75. Applying cERMIT on this data set leads to the results summarized in Table 1, where we assess our results with and without conservation in the context of a comprehensive recent comparison adapted from [21]. The species related to *Saccharomyces cerevisiae *used here were four yeast species in the *sensu stricto *clade, commonly used in other approaches relying on cross-species conservation.

**Table 1:** Benchmark comparison on 156 yeast ChIP-chip datasets

Motif finder	Number of successes top 1
AlignACE	16
MEME	35
	
MEME-c	49
Kellis	50
Converge	56
PRIORITY-C	69
	
MD-scan	54
PRIORITY-DC	78
	
ERMIT	75
cERMIT	87

Comparison of cERMIT with other motif finders on the yeast dataset of 156 ChIP-chip experimental conditions corresponding to 80 unique transcription factors (adapted after [21]).

We observed a dramatic increase in terms of number of recovered motifs as compared to AlignACE and MEME, which make use of only *S. cerevisiae *genomic sequence information and do not exploit quantitative information on binding, or conservation across species. MEME-c, the Kellis approach [33], and Converge [32] are heavily based on conservation information across the four related yeast *sensu stricto *species, yet result in a substantially lower number of successfully predicted motifs even when we do not make use of conservation (ERMIT). We also improve significantly on MD-scan, which uses ChIP-chip information but no conservation. The recently introduced PRIORITY algorithm is a state-of-the-art Gibbs sampling approach that can utilize both conservation (PRIORITY-C) and ChIP data (PRIORITY-DC), the latter by calculating discriminative counts obtained from bound versus unbound probes [21]. Even P RIORITY-DC produces a smaller number of successful predictions than cERMIT, and overall, the performance improvement compared to other recent approaches is significant. Another recent assessment of motif finders also included results on this yeast ChIP dataset. The assessment was part of the description of Amadeus [25], a motif finding platform that introduces multiple strategies for detecting enriched motifs, based on ranking all genes by the evidence of binding. The gold standard defined in this paper was highly similar to the set in Table 1. We extracted the intersection set between the two datasets [10,25], which contained 150 experiments (77 TFs). In contrast to the more stringent evaluation in [10], this study defined a success if any of four motifs (the top two predictions obtained by running the motif finder on fixed word lengths of eight and ten nucleotides) matched the known consensus. As cERMIT identifies motifs of flexible length, we compared the top four cERMIT predictions to the results reported in this study in Table 2. We used the results provided on the Amadeus website, which are based on the same similarity metric and on a threshold of 0.76, similar to the one used for the results in Table 1.

**Table 2:** Benchmark comparison on 150 yeast ChIP-chip datasets

Motif finder	Number of successes top 4
Trawler	52 (43)
YMF	57 (38)
AlignACE	64 (44)
MEME	76 (47)
Weeder	78 (53)
Amadeus	90 (63)
	
ERMIT	92 (61)
cERMIT	114 (66)

Comparison of cERMIT with other motif finders on the yeast dataset of 150 ChIP-chip experimental conditions corresponding to 77 unique transcription factors (adapted after [25]). In brackets are reported the number of unique transcription factors corresponding to the number of conditions recovered by each of the methods.

As can be seen in Table 2, results are consistently better when allowing for more than just the top scoring motif to be counted. Again, cERMIT showed a superior performance, and not just at one particular cutoff: The authors of Amadeus also reported their performance based on a cutoff of 0.82, at which they successfully recovered motifs for 78 conditions covering 53 TFs; cERMIT (100 conditions covering 58 TFs) clearly exceeds these numbers. We finally assessed cERMIT in comparison to DRIM, a recent motif finer that is likely the closest to our approach but was not part of the previous two comparisons [24]. While the DRIM manuscript also contained results on yeast ChIP-chip data, the authors considered a specific subset, not for all of which a known literature consensus is available. The subset of TFs with known consensus contains 44 conditions out of the set of 156 from Table 1, corresponding to 36 unique TFs. DRIM generally predicts more than one motif, with an average of 2.5 motif predictions per ChIP-chip dataset. For the purpose of a meaningful comparison, we verified whether the cERMIT results from Table 1 that relate to these TFs contained a successful prediction among the top two and three motifs. DRIM successfully predicts motifs for 26 conditions and 19 TFs (a 53% success rate on the level of TFs). cERMIT identifies the correct motif among the top two predictions in 30 out of the 44 conditions, and 30 of the 36 TFs (83%); for the top three motifs, these numbers increase to 32 conditions and 31 TFs.

#### Performance aspects

In addition to the empirical benchmark, an additional in-depth analysis of the performance of cERMIT teased apart its individual components in the context of the benchmark dataset. In particular, cERMIT was evaluated with regard to the choice of scoring function (using a range of pre-defined cutoffs on *P*-values instead of averaging); the contribution of the search over motif space compared to exhaustive enumeration of all 6-mers; and adding the evolutionary conservation filter.

In summary, these experiments show that averaging is the more robust option for a scoring function, in comparison to thresholding (see Materials and methods), which is more sensitive to noise and not consistent across different cutoffs. This is especially noticeable in the context of ChIP data without using conservation as filter to reduce noise. The search strategy significantly improves the performance relative to fixed length motif search, while a transformation of the ChIP-chip *P*-values into approximate Bayes factors (see Materials and methods) does not result in a significantly different performance.

In a further analysis, we examined how cERMIT's successful predictions agree with the other motif finders in two particular cases: when there is consensus among all other approaches on a successful prediction, and when no other approach manages to find the known literature consensus motif. As expected, cERMIT is able to find almost all known motifs of the first category. However, cERMIT is able to identify a substantial number of additional motifs (typically around 25% of its motifs) in the second case, and this is correlated with the use of conservation to increase the signal to noise. The complete details are reported in Additional file 1.

#### An assessment of false positives and false negatives

Through a permutation test, we can obtain significance estimates for the scores of the top cERMIT prediction (see Materials and methods section). This helps to investigate cases in which the motif search appears to fail, and to pinpoint experiments in which the scores for even the best predicted motifs do not rise above background scores on randomized data. To check the consistency of these estimates, we compared them with results from the motif finder PRIORITY, which also included a similar significance analysis [21]. In the following, we applied a stringent *P*-value cutoff threshold of 10^-4 ^to the cERMIT results summarized in Table 1. Comparing how estimated *P*-values agree with successful predictions - that is, the cases in which the top motif corresponded to the known literature consensus - we observed 67 true positives (TPs), 21 false negatives (FNs), 50 true negatives (TNs), and 18 false positives (FPs), which corresponded to a FP rate of 26% and a TP rate of 76%. On the FN side, where we fail to assign high enough significance to predictions that match the literature, we have 21 cases, and for 7 of these, PRIORITY also reported a non-significant match. This means that even when the signal from the experiment does not exceed random expectation, motif recovery may still be successful.

As we saw, there are also many cases in which the literature motif is among the top three or four reported motifs, but not at the top, and these cases somewhat misleadingly count as FPs here. We further investigated the top predicted motifs in these cases, where a significant *P*-value did not match the literature consensus of the factor assayed in the experiment. At least for eight cases (involving the TFs DAL81, INO4, MET32, MSN2, MSN4, and TEC1), there is convincing circumstantial evidence explaining the predictions. These cases are likely due to experimental conditions in which several factors regulate a largely overlapping set of target genes, and this effectively demonstrates cERMIT's ability to predict more than one functional motif in the same experiment. Details are given in Additional file 1.

Looking at the overall results from a different angle, there were only 34 conditions in which cERMIT (with or without using evolutionary information from other species) failed to recover the literature consensus motif among the top three predictions. When using conservation, 25 of these had comparatively large *P*-values (> 10^-4^), and may be cases in which the experimental noise may have been too high to successfully recover a functional site, or conditions in which the factor assayed does in fact not directly bind DNA. Furthermore, PRIORITY consistently did not assign a significant value and/or predict a matching motif for any of these. In the remaining nine cases with stringent *P*-values, three concerned the experiments INO4_YPD, TEC1_Alpha, and TEC1_YPD discussed in Additional file 1, which likely corresponded to cases where another protein in a complex is enriched, or in which the reported consensus is similar to the prediction but not called at the predefined threshold. We failed to report a high ranking matching prediction under any condition for only six TFs. Overall, this means that we are able to explain almost all ChIP-chip experiments. Contrary to the reportedly low success rates of various algorithms on the originally formulated motif finding problem [2], this shows that motif discovery on current genomic datasets has now become a highly successful undertaking.

#### Novel predictions

Finally, we ran cERMIT on the complete set of 352 experiments described by Harbison *et al*. [31] for 51 of the 196 datasets without known TF consensus, cERMIT predictions had a *P*-value > 10^-4^. The recent PRIORITY publication [21] reported predictions for a total of 82 out of the 196 experiments. Comparing our novel predictions to significant PRIORITY predictions provides computational support for predicted motifs from two highly different motif finding approaches. Out of the 82 PRIORITY predictions, 18 passed the stringent *P*-value cutoff of 10^-4^, while cERMIT passed this cutoff for 25 motifs out of these 82. Significant predictions overlap on 12 conditions, and the actual predicted top PSSMs were similar to each other in 7 out of the 12 cases. This shows a trend for the motif finders to agree on the top motifs if both are supported by stringent *P*-values. A comprehensive set of predictions obtained for experiments with and without available TF literature consensus are included in Additional file 1 with their corresponding significance.

### Identification of motifs from deep sequencing ChIP-seq experiments

ChIP-chip experiments are in the process of being replaced by ChIP-seq experiments, in which ChIP is followed by high-throughput sequencing of the bound DNA fragments. This allows for a cheaper and potentially less biased assay of the whole genome, but like genomic ChIP-chip before it, poses new challenges for motif finding, as the number of bound regions can be in the hundreds or even thousands. Not all motif finders are able to deal with input sets of such a large size efficiently, and some are not applicable at all. cERMIT has been specifically developed to make use of evidence for a genome-wide set of regulatory regions. For the compact yeast genome, ChIP experiments followed the common assumption that binding sites are found in close proximity to the open reading frame. The definition of an appropriate set of putative regulatory regions is a more difficult task in multicellular eukaryotes with more complex genomes. For instance, randomly selecting intergenic regions in mammalian genomes will include a large fraction of non-regulatory sequences such as repeats. However, high-throughput sequencing technology has already demonstrated its great promise for the study of gene regulation in such organisms, and we can resort to currently available experimental measurements of salient features of gene regulation at a whole-genome scale.

As our main focus is on condition-specific regulation, we would ideally define the search space to be the complete set of enhancer regions in the genome, or at least those active within the specific condition. In a recent paper [34], the authors mapped thousands of *in vivo *target sites of the enhancer-associated protein p300 using ChIP-seq, which provides a large set of enhancer regions conditional on interactions with p300. A perhaps even more comprehensive strategy for defining potential enhancer regions is to use regions known to fall within open chromatin, which tend to be accessible to binding by regulatory factors. This has been assayed, for example, by DNaseI digestion, and DNaseI hypersensitive sites (DHSs) have been determined by high-throughput sequencing [27].

**Figure 2:**
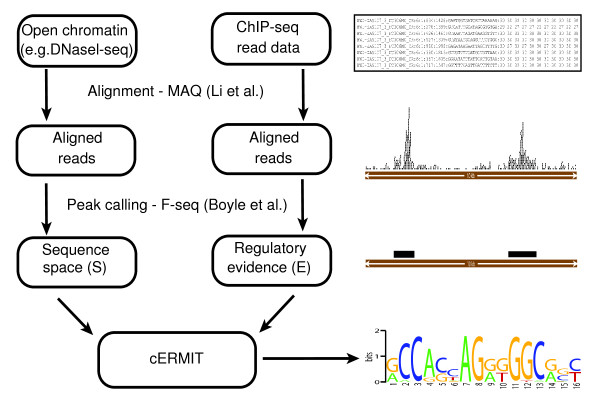
**Motif discovery pipeline**. Pipeline for motif discovery based on genome-wide evidence of regulation. Sequence reads are aligned to the reference genome and peak calling is executed to produce a set of putative regulatory regions (for example, DNaseI peaks) and corresponding evidence of regulation (for example, ChIP-seq peaks). As a final step in the pipeline, cERMIT is run on the preprocessed data to produce motif predictions that are best supported by the observed experimental evidence *E*.

Starting from such data in their entirety, we can then study the more nuanced transcription regulation signals that control condition-specific gene-regulatory programs. Hence, we utilize high-throughput deep sequencing information in two parallel ways: first from assays defining our space of putative regulatory regions - for example, those around DHS peaks; and second from factor-specific binding evidence based on the corresponding ChIP-seq data. A schematic pipeline that intersects different sources of high-throughput regulatory evidence for motif prediction is shown in Figure 2. Comprehensive ChIP-seq gold standard datasets like that of yeast [32] are not yet available, and we therefore applied cERMIT on a number of currently available mammalian datasets from human and mouse. For all experiments, we started from the deposited raw sequence reads, which we realigned to the genome to avoid dataset-specific biases; this allowed us to demonstrate the success of cERMIT as part of a generic pipeline.

**Figure 3:**
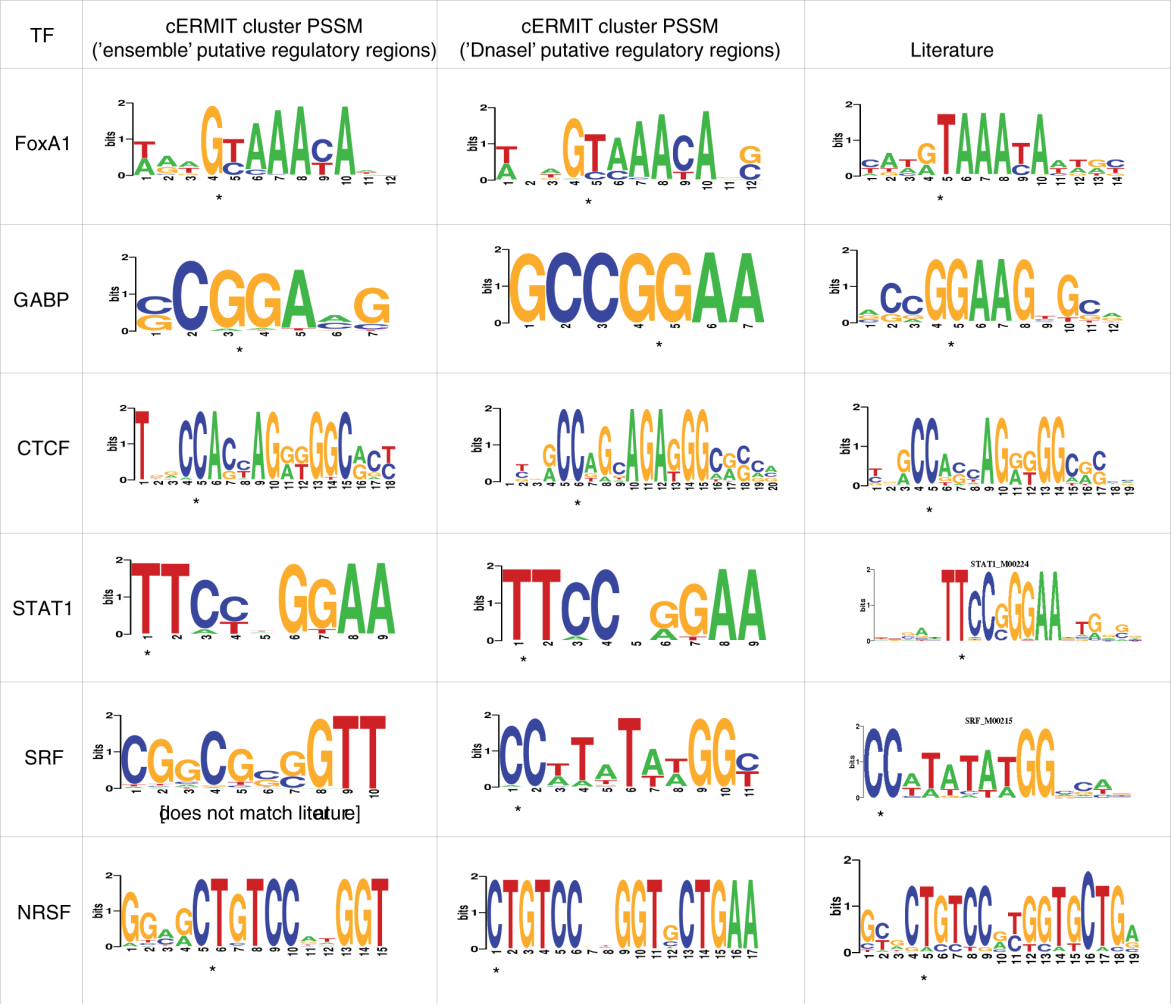
**Human ChIP-seq motif predictions**. Motif predictions of cERMIT on six human ChIP-seq datasets: STAT1 [35], the insulator binding protein CTCF [36], SRF, GABP [37], FoxA1 [38], and NRSF [39]. The 'ensemble' column includes results from using the ensemble of all six datasets to define the space of regulatory regions (see text). The 'DNaseI' column includes cERMIT predictions when using open chromatin regions, as defined by DNaseI peaks, to be the set of putative regulatory regions. Literature position-specific scoring matrices (PSSMs) were extracted from TRANSFAC 2009.1. Asterisks indicate the optimal alignment of motif prediction to literature. CTCF, due to its ubiquitous binding, was recovered using the top 25,000 DNase peaks as input to cERMIT. All other datasets consider the top 5,000 peaks from each factor (in the two different scenarios).

We first analyzed six human ChIP-seq datasets on factors STAT1 [35], the insulator binding protein CTCF [36], serum response factor (SRF), GA binding protein (GABP) [37], FoxA1 [38], and neuron-restrictive silencer factor (NRSF) [39]. Results from the cERMIT analysis are reported in Figure 3. We defined the space *S *of putative regulatory regions based on published human DHS data [27]. This definition was contrasted with an 'ensemble' approach, in which we took the combined set of high scoring peaks from a panel of ChIP-seq experiments and, after merging overlapping regions, arrived at one final set *S *used in common for the analysis of each individual factor. The evidence *E *was then assigned in factor-specific fashion, based on overlap of the commonly defined regions in *S *with the factor's ChIP-seq peak regions. The ensemble approach is an alternative especially suitable for conditions or species for which DHS data are not available: it is effectively an approximation to open chromatin regions, potentially under different experimental conditions depending on the particular ChIP-seq panel used, and provides a reasonable substitute for the DHS data as high scoring ChIP-seq peaks are known to be enriched within DHSs. We observed that the DNaseI approach worked extremely well in all six datasets in human. The ensemble approach resulted in similar performance, with the exception of SRF, which has relatively low enrichment of binding sites in ChIP-seq peak regions compared to the other factors. This seemed to result in too weak a signal to detect based on the whole ensemble of input regions.

**Figure 4:**
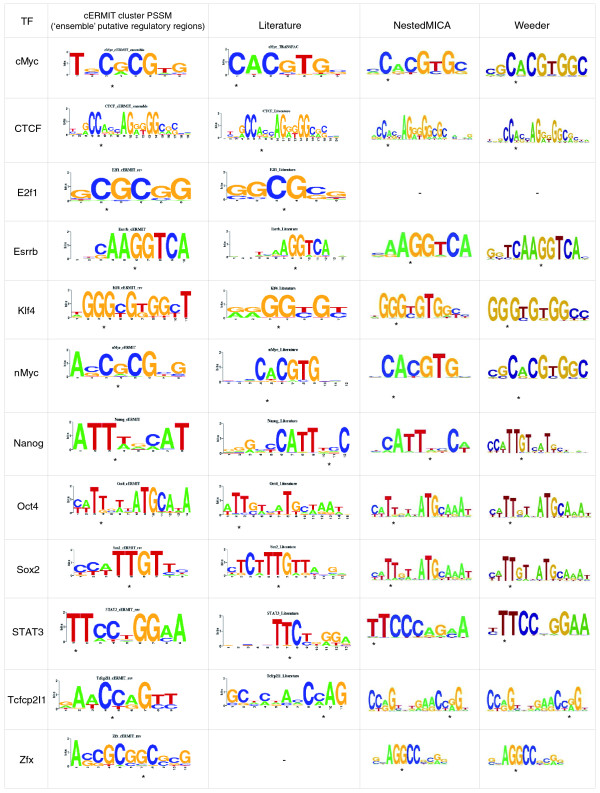
**Mouse ChIP-seq mouse**. Motif predictions of cERMIT on mouse ChIP-seq data from [40]. The predictions of cERMIT use the 'ensemble' approach to define the set of putative regulatory regions (see text for details). Literature position-specific scoring matrices (PSSMs) were extracted from TRANSFAC 2009.1, except for CTCF [45], Klf4 [57], and Zfx (unknown). Asterisks are used to indicate the optimal alignment of motif prediction to literature. Each individual factor contributes (the top scoring) 5,000 peaks to the ensemble set of putative regulatory regions.

The largest single mammalian ChIP-seq panel has been published as part of a study of TF binding in mouse embryonic stem cells [40]. We applied cERMIT to 12 datasets from this study: cMyc, nMyc, E2f1, CTCF, Esrrb, Klf4, Nanog, Oct4, Sox2, STAT3, Tcfcp2I1, and Zfx. As no DHS data have been published for mouse so far, we use the ensemble approach to define the set of putative regulatory regions. We included additional data for the non-sequence-specific factor p300 to define the space of regulatory regions, as its broad repertoire of binding partners should help to define an appropriate target set. Results from the cERMIT analysis are shown in Figure 4, which also shows the motifs identified in the original study, using the two popular algorithms Weeder [14] and NestedMICA [41]. In all cases cERMIT recovered a good approximation to the known literature binding specificity. For Zfx, there is no known literature consensus, and in that case cERMIT's prediction agrees with the results reported by the other motif finders. The E2F dataset was reportedly noisy, and no motif was reported by the other motif finders. While cERMIT successfully identifies a short GC-rich sequence motif resembling part of the site, it fails to expand to a longer motif matching the longer consensus (for example, as reported in JASPAR [42,43]). Finally, in the case of Sox2, cERMIT detected a more precise definition of each binding site than both Weeder and NestedMICA, whose prediction corresponded to motifs spanning sites for both Sox2 and Oct4, which are known to frequently co-occur as a module and co-regulate target genes. This demonstrates a strength of cERMIT as compared to Weeder and NestedMICA; it is able to integrate the quantitative evidence for tens of thousands of putative regulatory regions (35,500 regions for the mouse 'ensemble' set), rather than running on a small set of a few hundred highly scoring regions, in which a co-occurring motif might dominate over the true targets of the assayed factor. This makes the proposed motif discovery pipeline naturally suited to take full advantage of the state-of-the-art high-throughput sequence data.

### Application to microRNA transfection assays

ChIP is a direct assay of binding, and motif finders can be expected to work best in such a setting, which should deliver a good signal only for a set of direct true targets. However, knockdown or over-expression of regulatory factors followed by expression analysis is also common, and a rich data source for motif discovery. While these experiments also analyze the influence of a regulatory factor, this is done indirectly on the level of expression changes, and typically induce changes for direct targets containing functional sites, as well for indirect targets as a result of downstream effects.

As an example, we look at microarray expression data assaying gene expression changes upon induction of specific microRNAs. In particular, we evaluate recent data in which five microRNAs were considered: hsa-let-7b, hsa-miR-1, hsa-miR-155, hsa-miR-16, and hsa-miR-30a [44]. In Table 3, we compare the score of the top cERMIT motif with the best score of the same objective function, restricted to the canonical miRNA 'seed' matches (that is, the complementary sequence to positions 2-7, 1-7, 2-8, or 1-8 of each miRNA). As can be seen, the cERMIT results always delivered a motif with a higher score, demonstrating the success of our search strategy. With the exception of the let-7b experiment, for which the scores for canonical seeds were much lower than in the other experiments, the predicted motifs were slight variations of the seed matches. We also applied the method to the protein mass spectroscopy data for the same microRNAs, which then successfully recovered all five microRNA binding motifs. Thus, in agreement with the conclusions from [44], changes in mRNA expression is significantly linked to miRNA motifs in many cases, but at least for some miRNAs, the effect appears to be more pronounced on the protein level.

**Table 3:** MicroRNA overexpression motif predictions

	mRNA_32 hr				Protein			
		
	Seed motif	Score	ERMIT	Score	Seed motif	Score	ERMIT	Score
let-7b	**CTACCT**c	5.4	**GSCCCCS**	15.2	**CTACCT**c	9.1	**MTACCT**cw	9.7
miR-1	**ACATTC**c	13.8	**RCATTC**c	14.5	**ACATTC**c	8.5	wn**RCATTC**c	9.9
miR-16	g**CTGCTA**	7.4	wwg**CTGCT**	10.2	g**CTGCT**A	10.3	tg**CKGCTR**	11.6
miR-155	**AGCATT**a	12.6	**WGCRTT**a	13.6	**AGCATT**a	13.4	**GCATT**aw	15.2
miR-30a	g**TTTACA**	10.0	yg**TTTACR**	10.9	g**TTTACA**	7.5	wg**TTTACA**w	8.5

The letters in bold correspond to the canonical 6-mer microRNA seed (that is, the complementary sequence to positions 2 through 7). For each microRNA we report the best of four canonical 6- to 8-mer seed match scores based on the ERMIT objective function, and the corresponding motif prediction resulting from the motif search (data from [44]).

## Discussion

In the classic motif finding framework the search aims to identify overrepresented short patterns in a pre-defined subset *S'*⊂*S *(with S being the genome-wide set of regulatory regions), which is assumed to be enriched in functional motif occurrences. We present here the implementation and application of a new system for the identification of functional non-coding sequence motifs, which is applicable to the motif finding problem in an alternative definition, where each regulatory sequence in the whole set *S *is annotated with quantitative experimental evidence. This method circumvents the problem of having to define a sequence set enriched in *cis*-regulatory targets, and makes use of the additional information provided by quantitative evidence from current high-throughput experiments. Other recent approaches on related problems have worked within this rephrased definition; for instance, rank-based algorithms have been described to generate canonical motif descriptions for protein binding arrays [45,46]. The FIRE algorithm [47] could also be mentioned in this context, as it is based on the idea that the presence of an oligomer in regulatory regions is statistically dependent on a relevant phenotype of interest (for example, expression level or expression cluster membership).

Compared to some other rank-order based approaches, it is important to note that cERMIT incorporates the entire genome-wide evidence of regulation into the motif search. This is achieved through a carefully chosen objective function that provides a simple, yet effective quantitative measure for co-regulation of a set of sequences, without the need to define any cutoffs. The inspiration for this overall framework, and the particular function we used [29,30], draws from gene set enrichment analysis [28,48], in which the aggregate evidence of a predefined gene set, such as a functional pathway, is used to increase the power to detect differential gene expression. Our approach can be seen as an inverse to gene set enrichment analysis: instead of scoring a pre-defined gene set, we are looking for new optimal gene sets defined by a shared occurrence of a hidden sequence motif. The gene set enrichment analysis framework has attracted considerable attention, and other objective functions have been proposed that can be explored as potential alternatives for cERMIT.

Of key computational importance is the fact that our objective function is efficiently computable, which allows cERMIT to determine a putative motif enrichment quickly, making the proposed direct motif search strategy feasible. To score a given partition corresponding to a given consensus motif, cERMIT operates on a set of sequences via a fast search in a suffix array data structure, which enables the detection of potentially thousands of matches to a pre-specified k-mer in a large set of DNA sequences highly efficiently [49,50]. Hence, the overall runtime of the algorithm on a standard single processor workstation is on the order of a minute per typical run for the comprehensive set of upstream sequences for a yeast TF of interest, and 2 to 5 minutes for the approximately 35,000 regions (approximately 1 kb) from human ChIP-seq experiments. Instead of directly searching for over-represented short patterns in a pre-defined set of co-regulated sequences, we update our candidate for optimal partition by updating the corresponding consensus motif. Thus, we perform a search on the discrete space of IUPAC motifs, which is independent of the number of regulatory regions and scales logarithmically with the total length of the sequences in *S*.

We have demonstrated that this strategy makes cERMIT easily scalable to genome-wide technologies such as ChIP-seq, which provide data for the analysis of a much larger sequence space for putative TF targets. While cERMIT does not require an explicit background model, it detects enriched motifs by virtue of analyzing their occurrence patterns in the complete set of regulatory regions. In higher organisms with a complex non-coding genome, the definition of regulatory regions is non-trivial; however, recent high-throughput approaches to map open chromatin, or factors such as p300 that interact with a range of enhancers, provide a good approximation. In fact, we could show that even a simple joint set of target regions from a panel of different TFs can serve that purpose, but this will lead to differences in performance if the TFs have a wide range in the number of biological targets. Data on open chromatin under different conditions is expected to increase through efforts of the ENCODE consortium [51]. As our results show, the definition of putative regulatory regions is already very good given the current limited data, even though the conditions of DNaseI-chip and ChIP-seq matched for only some experiments.

Our use of IUPAC consensus motifs occasionally results in underestimating the motif degeneracy; the PSSMs shown above are built in a post-processing step based on the consensus sequence in the predicted set of bound genes. Together with the objective function currently used by cERMIT, which searches for the oligomer most strongly associated with the evidence provided, this means that our results should not be considered as quantitative models of actual binding affinity, but rather as the core of a functional motif. In addition, similarly to others before us [52], we base our motif search procedure on the assumption that the experimental setup ensures sufficient concentration of the factor in order for it not to be a limiting step in the sequence binding reactions. This allows us to currently approximate the inherently stochastic DNA-TF interaction by modeling it as a binary event.

These items provide the scope for improvement, both in terms of a more flexible motif description as well as on the motif search strategy, by means of a stochastic search in place of the greedy approach. We expect that this will allow us to pick up more degenerate signals and to provide more quantitative models of the recovered functional sites. Instead of merely defining genome partitions by the presence of motifs, a probabilistic framework based on a joint likelihood as well as a formal model of uncertainty would also allow for the simultaneous inference of a motif model and the most probable set of target genes. We can also incorporate different types of sampling moves that will enhance our ability to explore the motif search space. This may allow us to better capture motifs with two half-sites separated by highly degenerate spacer regions, or combinations of two or more motifs. To that end we can consider defining partitions based on high scoring motifs that co-occur in regulatory sequences. Ultimately, our aim is to approach the harder problem of detecting combinatorial interactions of different factors that distinguish between biological states, be it between different tissues, specific developmental stages, or normal versus cancer conditions.

## Conclusions

Motif finding with an objective function based on genome-wide evidence of regulation provides a flexible and successful framework to integrate sequence data with high-throughput binding or expression information. In particular, we present a flexible and successful pipeline to analyze regulatory information resulting from applications of next-generation sequencing technology. We also demonstrated the usefulness of integrating high-level information on the genome-wide set of regulatory regions (such as defined by DHSs), with quantitative data on the genome-wide affinity of individual regulatory factors.

Together with other recent approaches that utilize available quantitative evidence of regulation, the results reported here demonstrate convincingly that motif finders that make intelligent use of this additional information consistently outperform earlier motif finders. In contrast to the notoriously difficult motif finding problem based on over-representation in sequence alone, the scale-up in genomic experimental techniques, combined with appropriate motif finders, has allowed for great progress on the problem of efficiently decoding the regulatory information in complex genomes.

## Materials and methods

In this section we provide a detailed description of the motif finding strategy implemented by cERMIT. Given a set of putative regulatory regions and a score representing evidence for regulation for each region, cERMIT searches for an optimal partition of the input sequence space into a positive and a negative set. The positive set consists of regions that have at least one occurrence of a candidate motif specified as IUPAC string. To search for the best candidate partition, an optimality criterion is used that reflects aggregate evidence of regulation for a set of sequence regions. A key assumption in the proposed procedure is that the evidence for regulation is large in the positive set and small in the negative set for only those partitions that are induced by functional motifs. The algorithm outputs a set of predictions for regulatory motifs that correspond to the optimal partitions found by our search strategy.

A putative motif set Σ = {*m*_1_,..., *m*_*T*_} is defined as k-mers over the alphabet of IUPAC symbols {A, C, G, T, W, K, R, Y, S, M, N}, where *T *is the number of k-mers in the set; typically, we consider k-mers of length 5-20. A sequence space of *d *putative regulatory regions is defined as *S *= {*s*_1_,..., *s*_*d*_} and in each region we are given an estimated evidence of regulation *E *= {*e*_1_,..., *e*_*d*_}. Each putative motif *m*_*j *_induces a partition of *S *into two sets: the positive set *S*^*j *^contains those regulatory regions in which *m*_*j *_is present and the negative set is the complement. A motif is considered present in a sequence region *s*_*k *_if an exact match to the IUPAC motif description occurs on either strand of a DNA sequence or on the forward strand of an RNA sequence. We denote the occurrence of motif *m*_*j *_in *s*_*k *_by *m*_*j *_∈ *s*_*k*_.

There are two essential components of the algorithm: an objective function to score evidence of regulation for a given k-mer and a search procedure that explores the motif space for high-scoring k-mers.

### Evidence of regulation

The statistical scoring of evidence of regulation in a sequence region will depend on the type of assay used to infer the binding specificity of factors. However, all the statistical scores we propose can be placed in the framework of log-odds ratios:(1)

where *e*_*j *_is the evidence for regulatory region *s*_*j *_and *f *denotes the likelihood of the experimental evidence for *s*_*j*_, given that it is in the positive set (*M*_1_) or negative set (*M*_0_).

The types of experimental evidence provided by the data discussed in this paper are *P*-values from ChIP-chip experiments, counts of aligned short sequence reads from ChIP-seq experiments, and expression changes from microRNA overexpression assays.

#### *P*-values as evidence of regulation: ChIP-chip data

For ChIP-chip data, *P*-values {*p*_1_,..., *p*_*d*_} provide binding evidence for *d *sequence regions in *S*. The *P*-value is an indication of evidence against a null hypothesis, in this case, that the sequence region is not bound. As such, using the *P*-value as a measure of the odds ratio of binding or as an error rate is a misinterpretation, often termed the '*P*-value fallacy'. In this context, a *P*-value of 0.001 does not directly correspond to an odds ratio of 1/0.001 = 1,000 for binding evidence. In [53] a simple calibration of *P*-values was introduced:

This calibration can be interpreted as an upper bound on the odds provided by the data for binding (*M*_1_) versus non-binding *M*_0_), the Bayes factor:(2)

where *π*(*θ*|*M*_*i*_) are the prior distributions for the *P*-values in the positive and negative set, respectively. In general, the Bayes factor is the ratio of the marginal liklihoods given the model-specific parameter priors of the two models. Under a parametric approximation to the distribution of *P*-values, *π*(*θ*|*M*_*i*_) ~ Beta(*θ*,1), the *P*-values in the negative set would be distributed uniformly, which corresponds to the value for *θ *= 1, while the positive set *P*-values could be assumed exchangeable with *θ *∈ (0,1). The following upper bound on *π*, derived in [53], is used to approximate the Bayes factor:

Returning to the example of a *P*-value of 0.001, the Bayes factor under the proposed correction calculates as 1/0.0188 ≈ 50. In the ensuing analysis we use *e*_*j *_= log[*B*_10_(*p*_*j*_)] as the evidence of regulation for region *s*_*j*_. A natural question to ask is how different this measure of evidence is from *e*_*j *_= -log[(*p*_*j*_)], and this is explored in the context of cERMIT in Additional file 1.

#### ChIP-seq reads as evidence for regulation

The counts of aligned short sequence reads in ChIP-seq experiments can be used to provide evidence of regulation. In [54] a kernel density estimator is used to score binding; this smoothes and normalizes the counts of aligned reads. For each sequence region *s*_*j*_, the maximum of the kernel density estimate over all locations in the sequence is considered as positive evidence (*M*_1_):

where *t *indexes positions in *s*_*j *_and *k*(*t*) is the kernel density estimate at position *t*. We also specify a background binding score *b*, which in this paper is taken as the 85th percentile, for all regions *s*_*j*_, of the strictly positive kernel density scores. The evidence of regulation for region *s*_*j *_is a log odds ratio:

#### microRNA over-expression assays as evidence for regulation

By microRNA over-expression, the change in expression of putative target mRNAs in the presence of a microRNA is quantified. Denote *X*_*ji *_as the expression measured for the *j*-th gene in condition *i*. The two conditions in our setting are *i *= 0 before microRNA over-expression and *i *= 1 after over-expression. A sequence region *s*_*j *_is the 3' UTR region for the *j*-th gene in the expression assay. The evidence of regulation for region *s*_*j *_is the log of expression fold-change:

### Integration of evidence: definition of the objective function

Given evidence *E *= {*e*_1_,..., *e*_*d*_} for a set of sequence regions *S*, a motif *m*_*j *_partitions *E *into a positive set *E*^*j *^where the elements of *E*^*j *^are the evidence for those sequence regions that contain motif *m*_*j*_, *E*^*j *^= *e*_*i*_: *m*_*j *_∈ *s*_*i *_for *i *= 1,... *d*}, the negative set is the complement.

We assume that there exists a 'true' motif m_* _that induces a partition of the evidence with a positive set E*. This partition can be recovered by searching over the discrete space of motifs using an appropriate objective function. This objective function should capture high aggregate evidence for regulation in the positive set and low evidence in the negative set. The number of candidate partitions over the set of sequences is very large (at most 2^*d*^, where *d *is the total number of sequence regions) so this objective function must be efficiently computable.

A test statistic introduced in [29,30] has the above properties and is used as the objective function for cERMIT. Given evidence *E*^*j *^induced by a motif *m*_*j*_, we define:(3)

where |*E*| = *d *and |*E*^*j*^| are the cardinalities of the total number of regulatory regions and those contained in the positive set, respectively. The resulting optimization problem is:(4)

where  is our best guess at the optimal binding motif m_*_.

The variance in Equation 3 will be very large if the cardinality of the positive or negative sets is large, and the variance in estimates of the aggregate evidence will be large. Thus, these k-mers result in unstable scores and are considered implausible candidate k-mers. For this reason, we constrain the set size to be in the range [*a*, b × |*E*|], where *a *= 20, *b *= 0.15 for all ChIP-chip and overexpression data and *a *= 100, *b *= 0.30 for the ChIP-seq data. These bounds are effectively the only user-defined cERMIT parameters, and were chosen to reflect the complexity of the search problem (that is, more regions are targeted in the mammalian ChIP-seq experiments). This results in the following objective function:

Instead of averaging evidence over all genes, an alternative strategy is to select the top genes:(5)

where *k *is a threshold or selection parameter and 1_*x*>*k *_is the indicator function of whether *x *> *k*. The advantage of *J *over *J*_*sel *_is that it does not require the extra selection parameter *k*. When comparing both approaches within cERMIT, the classic bias/variance trade-off in statistical modeling is observed (see Additional file 1).

### Search strategy

It is not computationally feasible to optimize the cERMIT objective function in an exhaustive search over the space of all potential motifs. Instead, we adopt a direct greedy search strategy that relies on local motif updates to construct candidate motifs. The combined set of regulatory regions is maintained in a suffix array data structure [49,50], which, at minor pre-processing cost, allows for virtually constant time search for all occurences of a DNA k-mer of interest. We start from all possible 5-mers as seed points (in the case of TFs, pooling reverse complements, hence *T *= 512). Given a motif *m*, we construct a candidate set of motifs by locally varying the length and the degeneracy of *m*. The extension move takes a k-mer as input and independently appends or prepends A, G, C, or T generating eight new (*k *+ 1)-mers. When reducing the length of a motif we truncate the motif by one letter on either side to produce two new candidate motifs. Truncation is restricted to motifs of length 6 or longer. The degeneracy move operates on a single position in the k-mer at a time to produce a new motif candidate. The following update rules are applied to each position *j *in motif *m*:

1. *m*[*j*] = *A *then three new k-mers are constructed with *m*[*j*] set to *M*, *R*, *W *respectively;

2. if *m*[*j*] = *C *then three k-mers are constructed with *m*[*j*] set to *M*, *S*, *Y *respectively;

3. if *m*[*j*] = *G *then three k-mers are constructed with *m*[*j*] set to *K*, *R*, *S *respectively;

4. if element *m*[*j*] = *T *then three k-mers are constructed with *m*[*j*] set to *K*, *W*, *Y *respectively;

5. if *m*[*j*] = *R*, *Y*, *S*, *M*, *K*, *W *then *m*[*j*] is set to *N*, unless j = 1 or j = |*m*|;

6. if *m*[*j*] = *N *then the k-mer is not updated.

For a k-mer with no degeneracies, these moves will generate 3*k *candidate k-mers. For a k-mer with double degeneracy in all positions, the move will generate k-2 candidate k-mers with the same double degeneracy in all but one, non-terminal position, which is set to *N*.

For each seed motif *m *the search algorithm applies the update rules and examines if they result in a higher motif score, in which case the highest scoring candidate is used in the following iteration. This procedure is repeated until the update rules cannot improve the motif score, resulting in a candidate for the best scoring motif evolved from the particular seed.

The result of the search is a set of motifs  and their corresponding scores . For each motif we construct a PSSM based on the empirical counts of occurrences of each of the exact instantiations in the set of predicted target regions.

### Post-processing

Many of the top scoring motifs will be very similar, varying by a few letters. For this reason we add a post-processing step that clusters similar motifs around 'cluster centers' defined to be distinct individual k-mers with maximum objective function score J*.

In the clustering procedure we use the Harbison metric (at 0.75 cutoff) [31] to compute similarity between two motifs. For motifs *a*, *b *of equal length *w *the distance *D*(*a*, *b*) is:(6)

where *a*_*i*, *L *_and *b*_*i*, *L *_are the relative frequencies of base *L *at position *i *for the PSSM motif descriptions of *a *and *b*, respectively. For motifs of differing lengths we define the following 'Harbison similarity score':

where *a*', *b*' correspond to all possible overlaps of between motifs *a*, *b *induced by shifts such that the minimum overlap length is six, unless the motif itself is only five nucleotides long. This metric is also used in [10].

Two motifs *m*_1 _and *m*_2 _are considered similar if:

1. The PSSMs of *m*_1 _and *m*_2 _have Harbison similarity score ≥ 0.75;

2. The motifs *m*_1 _and *m*_2 _co-occur in the same sequences significantly more frequently than expected by chance, as measured by the following *P*-value threshold:

where *S*^1 ^and *S*^2 ^are the positive sets for motifs *m*_1 _and *m*_2_. The set of co-occurring regions *S*^*co*-*occur *^are those regions where the motifs *m*_1 _and *m*_2 _are both present and separated by at most *τ *nucleotides; we set .

Then, given the set of redundant output motifs , the following procedure outputs a set of motif clusters {*R*_*i*_} and smaller indices corresponding to higher motif scores:

1. Initialize the cluster count: *n *= 1;

2. Find the top motif in the set *C*

3. Add m* and all other motifs in *C *similar to m* to *R*_*n*_;

4. Remove the set *R*_*n *_from *C*;

5. Update cluster count *n *= *n *+ 1;

6. Repeat steps 3 to 5 until *C *is empty.

Given a motif cluster *R*_*i *_we can compute a cluster PSSM by averaging the PSSMs of each cluster member weighted by its motif score. We use this cluster summary in Figures 3 and 4.

### Integrating conservation

Sequence conservation between related species can be used to help guide the motif search: Defining the positive set based on the motif presence across a set of species can help to increase the signal-to-noise ratio by eliminating false positive matches that occur in individual genomes. We followed the example of previous approaches that utilized pattern co-occurrence without relying on alignments [21,55]. In our case, we incorporated conservation by refining the positive set *S*^*j *^of regulatory regions in which *m*_*j *_is present. If orthologous regions are available for a given region in one or more of the other species, we remove from *S*^*j *^those regions where *m*_*j *_is not found in all orthologous regions. That is, rather than restricting the analysis to the subset of genes with clearly defined orthologs, we simply require that patterns must co-occur in available orthologs; in cases where no ortholog is defined, set membership is based on the occurrences in the species with experimental evidence of regulation. This strategy allows us to make use of the full dataset, and we otherwise follow the same motif search procedure, applied to the refined set *S*^*j*^.

### Significance evaluation

For the top representative motif predictions {*m*_*k*_} we provide a *P*-value using a permutation procedure. Instead of an explicit representation of the empirical null distribution as a histogram, a parametric Gamma approximation was used instead to accommodate outliers in the tail area of the distribution. For a motif *m*_ℓ _with score *J*_ℓ _the following procedure is used to compute its *P*-value:

1. Generate 1,..., Π permutations of the evidence *E*, .

2. For each *π *= 1,..., Π compute

3. From  fit a Gamma distribution using the Maximum Likelihood criterion,

4. The *P*-value is 1 - (*J*_*l*_).

### Data

#### Yeast ChIP-chip compendium

The 352 ChIP-chip S. *cerevisiae *datasets and the corresponding orthologous probe sequences were extracted as described in [10].

#### microRNA overexpression

All mRNA and protein expression datasets were used as provided by [44]. Log-fold changes of the mRNA or protein expression values were assigned as evidence of down-regulation to the 3' UTR regulatory regions. In the case of mRNA expression, changes were assayed at two different time points, and we chose the later point at 32 h and compared it to 0 h as reference timepoint. The 3' UTR sequence set contains sequences of genes with RefSeq IDs (version 26) and a confirmed stop codon. This set was further filtered to exclude regions that are too short (<30 bp) or too long (>10,000 bp).

#### Human and mouse ChIP-seq experiments

The six human TF ChIP-seq datasets were used as provided in the following papers: STAT1 [35], the insulator binding protein CTCF [36], SRF, GABP [37], FoxA1 [38], and NRSF [39]. The 12 ChIP-seq datasets analyzed by cERMIT, cMyc, nMyc, E2f1, CTCF, Esrrb, Klf4, Nanog, Oct4, Sox2, STAT3, Tcfcp2I1, and Zfx, were used as provided by [40]. The embryonic stem cell panel additionally included datasets for the factors Suz12 and Smad1, which we did not consider in our analysis. The former factor does not interact directly with DNA; the dataset for the latter contained reads of length 36 bp instead of the reported 26 bp, and successful alignment to the mouse genome was significantly impacted. Unfortunately, we were unable to resolve the issue with the authors.

#### Processing of deep sequencing reads

##### Alignment of reads to the human and mouse genomes

Sequences from ChIP-seq experiments were aligned to the human genome (hg18) and mouse genome (mm9) using MAQ [26]. The reads aligned to four or less locations were retained. Additional filtering was performed to remove single base pile-ups of sequences by removing all sequence locations where, within a 30-bp window, there are more than 10 sequences of which 70% map to a single base location. Finally, at locations with more than five tags, tag counts were trimmed to a maximum of five.

##### Peak calling

Discrete ChIP peak calls were identified from ChIP-seq data using the non-parametric kernel density estimation procedure implemented in F-seq [54] with the threshold parameter *t *set to 10. Based on the Fseq base-pair scores we assign to each peak the maximum kernel density estimation value across all locations within the peak. In lieu of providing a predefined list of regions that are generally over-represented in ChIP-seq data (mitochondrial DNA, ribosomal genes, repetitive regions) we discard all regions with extremely large F-seq scores (≥ 10). Short peaks are extended to be at least 100 bp in length and long peaks are trimmed to be at most 1,000 bp. The extension/trimming is proportional to the distance of the end of a called peak region from its exact maximum location. The DHS peak regions were called as described in [27].

##### Definition of putative regulatory regions and assignment of evidence

A main goal in defining the set of putative regulatory regions is to be enriched in functional binding sites for the factor of interest. Recent high-throughput sequencing technologies coupled with a DNaseI assay have clearly demonstrated that regions of open chromatin are highly enriched in functional DNA elements [27]. Hence, we define our putative regulatory region set to consist of the DHS and call this the 'DNaseI' approach to defining putative regulatory regions. Ideally, we would use DHS data combined with the factor-specific ChIP-seq data from the same cell type. The only published DNaseI dataset in human is for CD4+ cells [27], which matches only the CTCF ChIP-seq cell type. The human SRF, GABP, and NRSF ChIP-seq data are derived from a lineage-related cell line (Jurkat human T lymphoblast cell line), which can be expected to share many open chromatin regions with the CD4+ cells. The remaining human ChIP-seq datasets were derived from cell lines unrelated to CD4+ - human HeLa S3 for STAT1, and MCF7 cells (human breast adenocarcinoma cell line) for FoxA1.

For mouse, no published high-throughput DHS data are available at this time. Hence, we adopt a different, yet closely related, strategy to define the putative regulatory regions, which relies on the assumption that ChIP-seq peaks tend to fall within open chromatin regions. The top ChIP-seq peaks across all mouse datasets provide a set of open chromatin genomic regions, which (after merging of overlaps) is assigned ChIP-seq scores from each individual dataset. In addition to the data for each factor for which we run motif finding, we include data for the non-specific factor p300. We call this the 'ensemble' approach to defining putative regulatory regions.

## Abbreviations

bp: base pair; ChIP: chromatin immunoprecipitation; ChIP-chip: ChIP followed by hybridization to microarray; ChIP-seq: ChIP followed by deep sequencing; DHS: DNaseI hypersensistive site; FN: false negative; FP: false positive; GABP: GA binding protein; IUPAC: International Union of Pure and Applied Chemistry; NRSF: neuron-restrictive silencer factor; PSSM: position-specific scoring matrix; SRF: serum response factor; TF: transcription factor; TN: true negative; TP: true positive; UTR: untranslated region.

## Authors' contributions

SG, UO, and SM designed the motif discovery framework and participated in all the analyses. SG, UO and KJ pre-processed the yeast ChIP-chip data and performed the analysis. XD contributed to the yeast conservation analysis. SG and UO pre-processed the microRNA induction data and performed the analysis. SG, UO and AB designed the motif discovery pipeline based on high-throughput sequencing data, pre-processed the human and mouse ChIP-seq data and performed the analysis. SG, UO, and SM wrote the manuscript with input from the other co-authors. All authors read and approved the final manuscript.

## Supplementary Material

Additional file 1 Tables with comprehensive prediction results on the yeast ChIP-chip datasets with known literature binding motifs as well as novel predictions. Further information, including an implementation of the proposed algorithm and a detailed description of the ChIP-seq pipeline, is accessible online [58]. Click here for file
