# Supplementary material for: Evidence-ranked motif identification
Source: Genome Biol. 2010 Feb 15;11(2):R19. doi: 10.1186/gb-2010-11-2-r19 (PMC2872879; doi:10.1186/gb-2010-11-2-r19)
Supplement: Additional file 1 — Tables with comprehensive prediction results on the yeast ChIP-chip datasets with known literature binding motifs as well as novel predictions. Further information, including an implementation of the proposed algorithm and a detailed description of the ChIP-seq pipeline, is accessible online [58]. [file gb-2010-11-2-r19-S1.pdf]

Supplementary Information

Evaluation of different components of the cERMIT algorithm

In this section we provide detailed evaluation of cERMIT with regard to the scoring function only, i.e. on all 6-mers without further search; adding the search; and adding conservation. We contrasted these comparisons with using a range of pre-defined cutoffs on P values, i.e. with a thresholded scoring function instead of averaging. All analyses were performed based on the 156 yeast ChIP-chip datasets from [31] (see Table 1 in main text), reporting the top prediction and the subset of 150 datasets [25] (see Table 2 in main text), reporting the top 4 predictions. The similarity cutoff used was as described in the main text (Harbison similarity ≥ 0.75).

Table 1: Comparison of different settings of cERMIT: fixed motif size (6-mers)

Scoring function	top 1 prediction		top 4 predictions	
	with conservation	without conservation	with conservation	without conservation
log_pvalues_Ave	73/156	45/150	99/156	65/150
BF_Ave	69/156	47/150	96/156	66/150
BF_Sel_0.01	63/156	47/150	94/156	62/150
BF_Sel_0.005	66/156	47/150	90/156	62/150
BF_Sel_0.001	64/156	43/150	97/156	62/150

Table 2: Comparison of different settings of cERMIT: variable motif size

Scoring function	top 1 prediction		top 4 predictions	
	with conservation	without conservation	with conservation	without conservation
log_pvalues_Ave	84/156	76/150	109/156	94/150
BF_Ave	87/156	75/150	114/156	92/150
BF_Sel_0.01	89/156	65/150	112/156	88/150
BF_Sel_0.005	89/156	73/150	112/156	91/150
BF_Sel_0.001	91/156	62/150	110/156	90/150

Legend:
Ave = 'Averaging' scoring function
Sel = 'Selection' scoring function (Sel_0.01 implies p-value cutoff = 0.01)
BF = Bayes Factors

Performance evaluation on "easy" and "hard" cases

In this section we briefly report on the performance of cERMIT on ChIP-chip datasets from Table 2 in the main text, on which all motif discovery methods perform well and on those on which all methods perform poorly. There are 34 datasets on which all methods find the known literature consensus. Without using conservation ERMIT successfully recovers 30 out of the 34. When using conservation, cERMIT recovers 32 out of the 34. There are 54 datasets on which none of the motif finders recovers the known literature consensus. Without using conservation ERMIT successfully recovers 12(22%). When using conservation, cERMIT recovers 25(46%).

Yeast ChIP-chip Additional Results

When applying cERMIT to the yeast ChiP-chip data, there are several cases in which the literature motif is among the top three or four reported motifs, but not at the top (cf Tables 1 and 2). We further investigated the top predicted motifs in cases for which a significant p-value did not match the literature consensus of the factor assayed in the experiment. At least for eight cases, there is convincing circumstantial evidence explaining the predictions:

1. DAL81_RAPA: The second best prediction matches the literature; the top prediction seems to match a few TFs that are all involved in a similar process: response to nitrogen catabolism (GAT1, GZF3, GLN3, DAL80)
2. TEC1_BUT14: The second best prediction matches literature; the top prediction matches the STE12 motif, and the two are known to form a complex: the second best prediction in STE12_Alpha in turn matches the TEC1 site (this was also reported in [10])
3. TEC1_Alpha: the top prediction again matches the STE12 motif
4. INO4_YPD: the top prediction matches the INO2 consensus, a family member with a highly similar binding profile, at a similarity of 0.86 (similarity metric defined in the main text); the similarity to the INO4 site just falls below our threshold
5. MSN4_H2O2_Hi and
6. MSN2_H2O2_Lo: The second best predictions match the literature. The top prediction matches the literature consensus of YAP family members, and YAP1 is known to be active under oxidative stress.
7. MET32_SM: and
8. MET32_YPD: The second best predictions match the literature. The top prediction matches the literature consensus of CBF1, which is one of the known auxiliary co-factors involved in the transcriptional regulation of the sulfur amino acid pathway

Full Yeast ChIP-chip Prediction Results

cERMIT predictions on the 156 yeast chip-chip datasets from [31] that have known literature consensus

#	experiment	literature	prediction	Harb. similarity	cluster	p-value
1	FKH2_YPD	TTGTTTACC	TGTTTAC	0.98	1	0.0000
2	NRG1_H2O2Hi	CCCT	CCCTR	0.98	4	0.0000
3	MET32_SM	CCACAGTTT	CCACAGT	0.98	2	0.0002
4	MAC1_H2O2Hi	GAGCAAA	RYGAGCAA	0.98	1	0.0003
5	PHO4_Pi-	CNMACGTG	MACGTGGK	0.98	1	0.0000
6	RPN4_H2O2Lo	TTTGCCACC	TTTGCCMCC	0.98	1	0.0000
7	MAC1_YPD	GAGCAAA	GAGCAAA	0.98	1	0.0000
8	BAS1_YPD	GAGTCA	RGAGTCA	0.98	1	0.0000
9	STE12_BUT14	ATGAAAC	TGAAACR	0.98	1	0.0000

10	MBP1_YPD	ACGCGT	YNACGCGT	0.98	1	0.0000
11	RCS1_SM	AAMTGGGTGCAKT	GGGTGCA	0.98	1	0.0034
12	MBP1_H2O2Lo	ACGCGT	YRACGCGT	0.98	1	0.0000
13	STE12_Alpha	ATGAAAC	TGAAACR	0.98	1	0.0000
14	REB1_YPD	TTACCCGG	RTTRCCCG	0.98	1	0.0000
15	YAP7_H2O2Lo	TTAGTAA	MTTAGTAA	0.98	1	0.0000
16	STB5_YPD	CGG	CGGTGYT	0.98	1	0.0000
17	RAP1_YPD	WRMACCCATACAYY	ACCCA	0.98	1	0.0000
18	ACE2_YPD	ACCAGC	MACCAGC	0.98	1	0.0000
19	GAT1_RAPA	GATAA	GATAASRNA	0.98	1	0.0007
20	HAP5_YPD	CCAAT	RCCAATG	0.98	3	0.6319
21	HAC1_YPD	KGMCAGCGTGTC	CGTGTC	0.98	1	0.0015
22	SUM1_YPD	MTTTTGTGSCRCT	TTTGTG	0.98	1	0.0000
23	RCS1_H2O2Lo	AAMTGGGTGCAKT	GGGTGCA	0.98	1	0.0000
24	MBP1_H2O2Hi	ACGCGT	ACGCG	0.98	1	0.0000
25	HAP3_YPD	CCAAT	RRCCAAT	0.98	3	0.3282
26	STE12_YPD	ATGAAAC	TGAAACR	0.98	1	0.0000
27	HAP2_YPD	ATTGG	ATTGGNYNR	0.98	2	0.2250
28	STE12_BUT90	ATGAAAC	TGAAACR	0.98	1	0.0000
29	CAD1_SM	TTAGTAA	MTTAGTAA	0.98	1	0.0004
30	YAP7_H2O2Hi	TTAGTAA	TACTAAKNS	0.98	1	0.0000
31	RCS1_H2O2Hi	AMTGCACCCAKTT	TGCACCC	0.98	1	0.0000
32	FKH2_H2O2Hi	GGTAAACAA	GTAAACA	0.98	1	0.0000
33	FKH2_H2O2Lo	GGTAAACAA	AAACAAW	0.98	4	0.2752
34	HAP2_RAPA	ATTGG	CWSATWGG	0.98	1	0.0000
35	HAP5_SM	ATTGG	TSATTGG	0.98	2	0.0001
36	LEU3_SM	MRCCGGTACCGGCR	CCGGY	0.98	1	0.0000
37	BAS1_SM	GAGTCA	RGAGTCA	0.98	1	0.0100
38	GLN3_RAPA	GATAAGATAAG	GATAAGR	0.98	1	0.0000
39	DAL82_RAPA	GAAAATTGCGTT	ATTGCG	0.98	2	0.4618
40	SIP4_SM	YCGGAYRRAWGG	AATGGAG	0.98	1	0.0047
41	REB1_H2O2Lo	TTACCCGG	TTRCCCG	0.97	1	0.0000
42	MCM1_YPD	WTTCCYAAWNNGGTAA	TCCYAA	0.97	1	0.0002
43	ZAP1_YPD	ACCCTAAAGGT	TASCCTA	0.97	1	0.0072
44	YAP1_H2O2Lo	TTASTMA	TTASTMAKC	0.97	1	0.0000
45	INO2_YPD	GATGTGAAAT	ATGTGAAR	0.96	1	0.0000
46	TEC1_YPD	RGAATG	RGAATGY	0.96	4	0.0260
47	MET32_YPD	CCACAGTTT	KCCACART	0.96	2	0.0003
48	FKH1_YPD	GGTAAACAA	GTMAACA	0.96	1	0.0000
49	TEC1_BUT14	CATTCY	RCATTCY	0.96	2	0.0053
50	CIN5_H2O2Lo	TTACTAA	TTACRTMAK	0.95	1	0.0000
51	RCS1_YPD	AMTGCACCCAKTT	TGMACCC	0.95	1	0.0703
52	CBF1_YPD	RTCACRTGA	RTCACRTG	0.94	1	0.0000
53	CAD1_YPD	TTAGTAA	MTTAGTMA	0.94	1	0.0000
54	MSN2_H2O2Hi	MAGGGG	MAGGGGK	0.94	1	0.0000
55	HSF1_H2O2Lo	AGAANNTTCTAGAA	YTCTRGAA	0.94	1	0.0000
56	MET31_YPD	CCACAGTTT	GCCMCAST	0.94	1	0.0000
57	UME6_YPD	WGCCGCCGW	WTWGCCGC	0.94	1	0.0000
58	SKO1_YPD	TGACGT	ATGRCGT	0.94	1	0.0000
59	HSF1_H2O2Hi	AGAANNTTCTAGAA	YTCTRGAA	0.94	1	0.0000
60	RPN4_YPD	GGTGGCAAA	GGTKGCA	0.94	1	0.0332
61	REB1_H2O2Hi	CCGGGTAA	MSGGTAA	0.93	1	0.0000

62	CBF1_SM	TCAYGTGAY	TCAYGTG	0.93	1	0.0000
63	SWI4_YPD	TTTCGNG	TTTCGYG	0.93	1	0.0000
64	CIN5_H2O2Hi	TTACTAA	CTTACNT	0.93	1	0.0000
65	AFT2_H2O2Hi	AAAGTGCACCCATT	GCRCCC	0.93	1	0.0124
66	LEU3_YPD	MRCCGGTACCGGCR	KACCGG	0.93	1	0.0000
67	MET31_SM	CCACAGTTT	GCCACAK	0.93	1	0.0565
68	ADR1_YPD	GGRGK	AAGGNGK	0.93	4	0.6707
69	RPH1_H2O2Hi	CCTTAAGGGG	RGGGG	0.92	1	0.0031
70	GLN3_SM	GATAAGATAAG	AGATWA	0.92	3	0.0624
71	MSN2_H2O2Lo	MAGGGG	MRGGGGYG	0.92	2	0.0070
72	AFT2_H2O2Lo	AAAGTGCACCCATT	GCMCCCR	0.92	1	0.0000
73	SWI6_YPD	TTTCGNG	KTTCGYG	0.92	1	0.0000
74	GCN4_RAPA	ARTGACTCW	TGACTCA	0.91	1	0.0000
75	GCN4_SM	ARTGACTCW	TGACTCA	0.91	1	0.0000
76	GCN4_YPD	ARTGACTCW	RTGACTCA	0.91	1	0.0000
77	RTG3_H2O2Hi	GGTCAC	GTCMC	0.91	1	0.0000
78	CIN5_YPD	TTACTAA	MTTACNTMA	0.91	1	0.0000
79	UME6_H2O2Hi	WGCCGCCGW	TWRCCGC	0.91	1	0.0000
80	YAP1_YPD	TTASTMA	TWAGTMAKC	0.90	1	0.0000
81	SKN7_YPD	GGWCCRGCCAAAT	GSCCMG	0.90	1	0.0000
82	SWI5_YPD	YCAGCM	CCAGCMA	0.90	1	0.0000
83	SKN7_H2O2Hi	GGWCCRGCCAAAT	GSCSRG	0.90	1	0.0000
84	HAP1_YPD	CCGNTANNNCCG	KAWNNCCGA	0.89	3	0.0000
85	MSN4_H2O2Hi	MAGGGG	MRGGGG	0.89	2	0.0009
86	HAP4_YPD	YCNNCCAATNANM	RCCMATSA	0.89	1	0.0000
87	NRG1_YPD	AGGG	WAGGSWC	0.88	2	0.0161
88	SKN7_H2O2Lo	ATTTGGCYGGSCC	SGGSC	0.88	1	0.0000
89	STP1_YPD	RCGGCNNNRCGGC	CRCGGCA	0.88	1	0.0143
90	SUT1_YPD	CGCG	CRCGG	0.87	3	0.0000
91	MCM1_Alpha	TTACCNNSTTRGGAAS	TYRGGMAA	0.86	1	0.0000
92	RTG3_RAPA	GTGACC	GWGACYSR	0.86	1	0.0000
93	YAP3_YPD	TTACTAA	AWTACTA	0.84	4	0.9342
94	STP1_SM	RCGGCNNNRCGGC	YACGGCA	0.84	2	0.1886
95	YAP5_H2O2Hi	TTACTAA	TACTKARM	0.84	2	0.8989
96	RIM101_H2O2Hi	CTTGGCA	TTMGCAA	0.84	2	0.9989
97	ABF1_YPD	RTCAYTNNNNACGW	RTCACK	0.83	1	0.0000
98	DAL81_RAPA	AATCCCGCCCGCGGCTTTT	YRCGGC	0.83	2	0.0000
99	AFT2_YPD	AATGGGTGCACTTT	GAAAGGG	0.82	2	0.5781
100	DAL81_YPD	AATCCCGCCCGCGGCTTTT	CACGGC	0.82	1	0.0000
101	AZF1_YPD	AAGAAAAA	YAGARAA	0.82	2	0.0637
102	DAL82_SM	AACGCAATTTTC	CTTTTC	0.82	3	0.2101
103	RPH1_YPD	CCTTAAGGGG	TAAGASG	0.81	3	0.6215
104	RLM1_YPD	CTAWWWWTAG	CTAGW	0.81	3	0.2052
105	YAP6_H2O2Hi	TTACTAA	CCTAA	0.79	1	0.0000
106	YAP6_YPD	TTACTAA	CCTAA	0.79	1	0.0000
107	GAL4_GAL	CGGNNNNNNNNNNNCCG	CGGSG	0.79	2	0.9220
108	RTG3_SM	GGTCAC	AGTCA	0.79	1	0.0685
109	TYE7_YPD	CANNTG	CACSWGWY	0.79	1	0.0000
110	DAL80_RAPA	GATAA	GTTAAMAA	0.79	4	0.3117
111	GAT1_SM	GATAA	GTTAAAA	0.79	3	0.9728
112	YOX1_YPD	YAATA	WAWTAGGA	0.78	4	0.8999
113	NRG1_H2O2Lo	AGGG	RGGSAC	0.78	3	0.0012

114	GCR1_YPD	GSGGAAGCC	ASTSGAA	0.78	4	0.0243
115	MSN2_Acid	MAGGGG	MACGG	0.77	4	0.0000
116	MSN4_YPD	CCCCTK	CCCAK	0.77	1	0.0112
117	PUT3_SM	CGGNNNNNNNNNNCCG	MYCCCGA	0.75	1	0.0011
118	YHP1_YPD	CAATTA	TWASTGT	0.74	4	0.8391
119	DAL80_YPD	GATAA	TAGMTRSA	0.73	2	0.9500
120	GAL4_RAFF	CGGNNNNNNNNNNNCCG	GAWCCG	0.73	3	0.5424
121	MSN4_Acid	MAGGGG	MGGGC	0.72	3	0.0000
122	ARR1_YPD	TTACTAA	STTAMAA	0.72	4	0.8098
123	INO4_YPD	ATTTCACATG	YTCACAYG	0.71	1	0.0000
124	SMP1_YPD	ACTACTAWWWWTAG	GCTMC	0.71	2	0.4196
125	XBP1_H2O2Lo	CTCGAAG	CCSCG	0.70	4	0.1113
126	HAP4_H2O2Lo	KNTNATTGGNNGR	RAKATTGR	0.69	3	0.9999
127	MOT3_SM	YAGGYA	AGGTT	0.69	2	0.0044
128	ROX1_YPD	YSYATTGTT	CCTTG	0.69	2	0.0636
129	ASH1_BUT14	YTGACT	MGGAC	0.69	4	0.0000
130	YAP6_H2O2Lo	TTACTAA	MTTACMT	0.68	2	0.0001
131	ROX1_H2O2Lo	AACAATRWR	SATGSAA	0.68	4	0.0050
132	PUT3_YPD	CGGNNNNNNNNNNCCG	CGGGAWG	0.68	1	0.0006
133	PHO4_YPD	CNMACGTG	YWAAGT	0.68	2	0.5023
134	MSN2_RAPA	CCCCTK	WATMCCTT	0.68	1	0.9966
135	GZF3_RAPA	CTTATC	RTTAACA	0.68	4	0.3964
136	GLN3_YPD	CTTATCTTATC	GGTTKAG	0.68	3	0.1965
137	MOT3_YPD	TRCCTR	TTKCATAA	0.67	3	0.8480
138	ADR1_HEAT	MCYCC	ACYCGA	0.66	1	0.5249
139	PDR3_YPD	TCCGCGGA	CGRGTAAS	0.66	2	0.9938
140	UGA3_SM	CCGNNNNCGG	WMAYGGTA	0.66	2	0.9954
141	SIP4_YPD	YCGGAYRRAWGG	GATAGA	0.66	1	0.9984
142	RTG1_SM	GTGACC	WWGCCCA	0.62	3	0.7081
143	RTG1_RAPA	GGTCAC	GTAMACT	0.62	3	0.9899
144	DAL82_YPD	AACGCAATTTTC	ACAGSAM	0.62	4	0.3856
145	PDR1_H2O2Lo	CCGCGG	AGKCGG	0.61	1	0.9982
146	GAL4_YPD	CGGNNNNNNNNNNNCCG	TCCGMAS	0.61	3	0.5648
147	YAP5_YPD	TTAGTAA	SKTAATT	0.60	4	0.0084
148	RPH1_SM	CCTTAAGGGG	ACTKAG	0.60	3	0.8246
149	GZF3_H2O2Hi	CTTATC	KTACARG	0.60	1	0.6396
150	TEC1_Alpha	RGAATG	GAGAAAA	0.58	2	0.0137
151	UGA3_RAPA	CCGNNNNCGG	TATMCTG	0.54	1	0.0058
152	MSN4_H2O2Lo	MAGGGG	YTAAGMG	0.54	3	0.3631
153	PDR1_YPD	CCGCGG	AWGTGGG	0.52	2	0.0318
154	RTG3_YPD	GTGACC	ARYAYCAS	0.50	3	0.7510
155	ROX1_H2O2Hi	AACAATRWR	CCGAM	0.46	1	0.0000
156	MSN4_RAPA	MAGGGG	AMCWTAG	0.45	4	0.9965

Novel Predictions

cERMIT predictions on the 196 yeast chip-chip datsetset from [31] that do not have known literature consensus.

#	experiment	prediction	p-value
1	CRZ1_YPD	SGGSC	0.0000

2	MOT3_H2O2Hi	GGRGG	0.0000
3	RGM1_YPD	ATCASNAC	0.0000
4	ARG80_SM	MCSGG	0.0000
5	STB1_YPD	WTTTGGCG	0.0000
6	FHL1_YPD	GYAYRGRTK	0.0000
7	FHL1_H2O2Hi	CCRKRCM	0.0000
8	SKN7_HEAT	CYGGSSC	0.0000
9	YDR026c_YPD	KTTAMYMGG	0.0000
10	RAP1_SM	KGYRYGG	0.0000
11	FHL1_RAPA	GYMYRGRKK	0.0000
12	DAL81_SM	SGGSC	0.0000
13	SNT2_YPD	GNYAGCGCC	0.0000
14	PHO2_H2O2Hi	ASGGG	0.0000
15	ASH1_YPD	SGSCG	0.0000
16	RFX1_YPD	TTRCYATG	0.0000
17	FHL1_SM	AYCYRNRCA	0.0000
18	MTH1_GAL	SGGSC	0.0000
19	XBP1_YPD	GCKGNG	0.0000
20	SFP1_SM	GTAYKGRY	0.0000
21	SOK2_BUT14	SCTGCR	0.0000
22	MET4_YPD	CCACRSYT	0.0000
23	TOS8_YPD	CMCNGC	0.0000
24	DOT6_YPD	SGGSC	0.0000
25	MTH1_YPD	SMWGGC	0.0000
26	DIG1_Alpha	YGTTTCAR	0.0000
27	GAT3_YPD	GTRNKGRT	0.0000
28	OPI1_YPD	TYCACATG	0.0000
29	SOK2_YPD	WTARNSTTT	0.0000
30	DIG1_BUT90	MTGAAAC	0.0000
31	SFP1_YPD	GSMAGA	0.0000
32	NDD1_YPD	RNGTMMACA	0.0000
33	SPT2_YPD	KRTMCAAMT	0.0000
34	RPN4_H2O2Hi	GGTRGMAMAW	0.0000
35	PHD1_YPD	RGGYAC	0.0000
36	GAT1_YPD	GCGRSS	0.0000
37	DIG1_BUT14	TGMRACRC	0.0000
38	MAL33_YPD	GGTGS	0.0000
39	PHD1_BUT90	RSGCAC	0.0000
40	DIG1_YPD	TGMAACANR	0.0000
41	MSN2_HEAT	TWTCTWGMA	0.0000
42	MAL13_YPD	GRKKTSGG	0.0000
43	RIM101_YPD	CWTCAA	0.0000
44	STP4_YPD	GCCSYAC	0.0000
45	RLR1_YPD	TGTANANTA	0.0000
46	GCR2_SM	AATAGARS	0.0000
47	DAT1_YPD	MYCACCAY	0.0000
48	MIG1_GAL	CRGKKG	0.0000
49	MET4_SM	SMTGAMTC	0.0000
50	ARG81_SM	GAGTCAM	0.0000
51	RGT1_YPD	TTTKTMCGR	0.0001
52	MSN2_YPD	YYKANTAAC	0.0001
53	THI2_Thi-	TTAGRRC	0.0007

54	CHA4_SM	TGNNTGGGW	0.0011
55	ARO80_YPD	CKCGGYA	0.0014
56	HIR2_YPD	RTGCGAA	0.0023
57	HMS2_YPD	TGCARGAW	0.0025
58	IXR1_YPD	TGCRTRRG	0.0028
59	ARO80_SM	AKTGMCG	0.0028
60	HSF1_YPD	KTSTRGAMM	0.0029
61	ARG81_YPD	TGASTCMY	0.0031
62	HOG1_YPD	MTWWMCAAKR	0.0033
63	PHO2_SM	GCATGRS	0.0035
64	ECM22_YPD	MGGKGS	0.0038
65	RGT1_GAL	KTTYTCCG	0.0060
66	GTS1_YPD	KAMTAWC	0.0077
67	GCR2_YPD	TAAGTWSK	0.0101
68	CUP9_YPD	TGACGAR	0.0141
69	MGA1_H2O2Hi	MGMNCRG	0.0156
70	KSS1_YPD	TWTMTTRKC	0.0202
71	YFL044C_YPD	AWTRATACR	0.0236
72	MET18_YPD	STAAGTT	0.0267
73	YAP1_H2O2Hi	ATCYGAMA	0.0269
74	HIR1_YPD	ACCGKAY	0.0293
75	ARG80_YPD	GAGTYMMA	0.0300
76	PHD1_BUT14	WAGGGYWRC	0.0338
77	HIR3_YPD	ATGTMYGG	0.0474
78	RME1_YPD	WRCCCTMA	0.0557
79	RTG3_H2O2Lo	GTCANGC	0.0675
80	SFP1_H2O2Lo	KRWSACATT	0.0677
81	PHO2_Pi-	MCCACST	0.0689
82	YML081W_YPD	TCRTMAWA	0.0809
83	YPR196W_YPD	CAMATGC	0.0836
84	STB4_YPD	ATCCGAR	0.0964
85	YDR520C_YPD	AACCCKC	0.1627
86	YJL206C_H2O2Lo	MKAYMCAWG	0.1697
87	KRE33_YPD	GTTCAAY	0.1778
88	YJL206C_YPD	GTKGGGG	0.1796
89	OAF1_YPD	CGCRCYY	0.1939
90	FZF1_YPD	AGATGCM	0.1985
91	YBL054W_YPD	TMAKYGCG	0.2238
92	KSS1_Alpha	TATGCAWT	0.2296
93	YLR278C_YPD	TCAMTA	0.2421
94	HMS1_YPD	TTATSYCA	0.2484
95	MAL33_H2O2Lo	TTGRWAY	0.2510
96	MET28_SM	AWGGTGG	0.2550
97	SRD1_YPD	ATTTRCARM	0.2558
98	YAP1_HEAT	RTRTACAMS	0.2750
99	TBS1_YPD	TAMKGTAY	0.2765
100	WAR1_YPD	TAAYYCWA	0.3132
101	MSN1_YPD	ATGRCCA	0.3317
102	SFP1_H2O2Hi	MRAAYATC	0.3435
103	YPR022C_YPD	TGAWAGT	0.3456
104	FAP7_YPD	SAGWGTA	0.3557
105	MIG1_YPD	TTAYGTAAK	0.3715

106	PIP2_YPD	WCAAGCNA	0.3782
107	YER051w_YPD	YCRMAKT	0.3931
108	MAL33_H2O2Hi	GANCAGG	0.3953
109	BYE1_YPD	WYGNARRTAT	0.4066
110	RPI1_YPD	RCGTARA	0.4182
111	YKR064W_YPD	AYGTGTMA	0.4223
112	UME1_YPD	WTARCCKC	0.4323
113	YBR239c_YPD	WCAATT	0.4435
114	PHO2_YPD	CMATCM	0.4558
115	STP2_YPD	TATAYARTA	0.4832
116	MOT3_H2O2Lo	RAAKCKGT	0.4849
117	RIM101_H2O2Lo	TCRWAG	0.5093
118	CST6_YPD	TKACAWAAA	0.5286
119	UGA3_YPD	MMTATGTA	0.5351
120	SFL1_YPD	TRKGSAAT	0.5497
121	YNR063W_YPD	AWACTATM	0.5613
122	RLM1_BUT14	ARRGYSAMAA	0.5802
123	YFL052w_YPD	RANCATTT	0.5868
124	STB6_YPD	ATRCAARAA	0.5984
125	MGA1_YPD	AYSAAAAG	0.6130
126	RTG1_YPD	ATMCYCR	0.6282
127	GAL80_YPD	ATGACAG	0.6495
128	MATA1_YPD	TRCWTAGW	0.6711
129	WTM2_YPD	MTMWMCRTA	0.7092
130	GAT1_HEAT	GSYMSSGS	0.7200
131	HAP4_SM	GGGWGAT	0.7294
132	RDR1_YPD	AWACTATA	0.7309
133	GAL3_YPD	ATTKAYAGW	0.7386
134	MIG3_YPD	WYMCAAYTT	0.7439
135	SPT23_YPD	TGTWMGGA	0.7556
136	CAD1_H2O2Hi	KTACTART	0.7603
137	SIG1_YPD	TTTRSCCSG	0.7759
138	WTM1_YPD	ATGWTAAT	0.7777
139	ARR1_H2O2Hi	WTCAWGTA	0.7853
140	IME1_H2O2Hi	SGAMRCWT	0.8071
141	MET28_YPD	ACATCAYY	0.8076
142	EDS1_YPD	AGRTASC	0.8140
143	RPH1_H2O2Lo	RTCTGARA	0.8232
144	HAA1_YPD	TGTAARTW	0.8322
145	MSS11_BUT14	TAATMCAW	0.8344
146	RDS1_H2O2Hi	CGRCMGA	0.8500
147	ADR1_SM	AAKRYAGYM	0.8632
148	YER184C_YPD	SSGGYYAAA	0.8731
149	YAP3_H2O2Hi	GWGGCGA	0.8807
150	RDS1_YPD	AATACRRA	0.8838
151	PHO2_H2O2Lo	TGYCCGA	0.8840
152	YRR1_YPD	WACGNGTRA	0.9223
153	IFH1_YPD	TATGCAWT	0.9238
154	PDC2_YPD	ATTRANGA	0.9249
155	THI2_YPD	ATCYTKRA	0.9328
156	MBF1_YPD	SCACAMAW	0.9375
157	HAL9_YPD	AARTCAG	0.9410

158	YDR049W_YPD	RRCACAAAA	0.9418
159	SMK1_YPD	STCAMCC	0.9457
160	NNF2_YPD	GMYTKARAA	0.9487
161	MDS3_YPD	KAWYGAA	0.9489
162	ZMS1_YPD	KTAATTA	0.9617
163	RCO1_YPD	AMRTGATG	0.9629
164	IME4_YPD	KTACTTMT	0.9687
165	YJL206C_H2O2Hi	AAAWGYTC	0.9701
166	UME1_H2O2Hi	AACTYAC	0.9716
167	YGR067C_YPD	AMACATTW	0.9720
168	GZF3_YPD	AATGYSAT	0.9726
169	YAP7_YPD	TAYNTACA	0.9796
170	YDR266c_YPD	GGKTRATM	0.9802
171	SUT2_YPD	GTAAKGY	0.9809
172	SIP3_YPD	TYTTSAA	0.9826
173	USV1_YPD	TNGCGGCT	0.9864
174	SIG1_H2O2Hi	TACSAAT	0.9897
175	MIG2_H2O2Hi	AMWACTAT	0.9903
176	IME4_H2O2Hi	TGTTRSAA	0.9909
177	CHA4_YPD	TTAKGGC	0.9921
178	RTS2_YPD	WMRCGATG	0.9925
179	IME1_YPD	TCSWCAAM	0.9925
180	MSS11_YPD	TMGATWSA	0.9930
181	UPC2_YPD	CARKATT	0.9936
182	ASK10_YPD	AAGTCRK	0.9944
183	MIG2_YPD	AAKTTCAA	0.9946
184	NDT80_YPD	RSCCGARW	0.9956
185	KSS1_BUT90	MGRTGWRYT	0.9962
186	STB2_YPD	ACCCMAC	0.9967
187	YER130C_YPD	RRCACAAAA	0.9985
188	SNF1_YPD	AATAWACAA	0.9986
189	ABT1_YPD	GAWAGTA	0.9987
190	YBR267W_YPD	WKGTRARTA	0.9990
191	SPT10_YPD	ATGTCMG	0.9990
192	PPR1_YPD	TTACCCSMM	0.9996
193	HSF1_HEAT	GTTAGC	0.9997
194	YKL222C_YPD	MCRKGTAWA	0.9997
195	ACA1_YPD	CCARTCA	0.9997
196	PUT3_H2O2Lo	TAGAKAC	0.9997
